# Proceedings of the Tennessee Dental Society

**Published:** 1879-02

**Authors:** 


### PROCEEDINGS OF THE TENNESSEE DENTAL SOCIETY.

*Held at Nashville, June 26th, 1878.*

Meeting convened according to adjournment, at 10 o'clock, with President Noel in the chair. Minutes of former meeting read and approved.

Dr. R. R. Freeman and H. W. Morgan, were appointed a committee on membership.

The committee on subjects for discussion reported the following which was adopted:

1. Dental Physiology.
2. Operative Dentistry.
3. Materials for Filling Teeth.

4. Mechanical Dentistry.
5. Dental Literature,
6. Microscopy.
7. Dental Appliances.
8. Anæsthetics.

J. C. Ross and J. A. Arrington, committee.

Dr. J. H. Powell, of Madisonville, Ky., made application for membership, and was unanimously elected.

Tho subject of Dental Physiology was presented for discussion.

Dr. Noel stated that he regarded the subject as one defining the use and natural functions of organs, claiming it to be the highest aim of the profession to preserve the teeth in good condition. The best means of securing this result is to observe perfect cleanliness, describing his method of securing this. He gave the chemical analysis of healthy and unhealthy saliva, claiming that destruction of the teeth is the result of acidulated saliva, principally acetic acid, generated by the decomposition of vegetable food. The principal acids generated by the decomposition of food is acetic, lactic, sulphuric and nitric. Chloric acids being formed by the use of condiments.

Dr. Freeman said the remarks of the president reminded him that there was a large field for vigilant work. Mental work as well as physical. Experience had convinced him that better results were secured by proper cleanliness than by fine operations neglected. If we will aid and assist nature in the performance of its functions, we have secured to our patients one of the most lasting benefits.

Dr. Prothro said he agreed with what had been said, but while nature will accomplish wonders, we can not depend on our patients to observe such strict habits of cleanliness as to secure the best results, but he resorted to free separations so as to force cleanliness by the natural performance of the function of mastication.

Dr. Beach—However much we may urge the necessity for cleanliness, the sequel shows an utter disregard in many instances of our good advice, hence, different individuals have to be differently treated. The occlusion of the teeth as nature has arranged them, should be also considered before separations are resorted to. In many cases separations are highly beneficial, when in others they are a positive injury. Teeth are not in a physiological condition in their relation to the mouth when they are free from caries. Irregular arrangement must be considered a departure from nature and demands treatment of the most skillful kind. Many mouths are changed from a physiological to a pathological condition by the want of proper care during the shedding of deciduous teeth, and whilst many good men in the profession shrink from the arduous work of correction of irregularities, there are thousands who ignore it altogether. No mouth can be in a physiological condition with teeth crowded out of their natural position for any length of time.

Dr. Noel called the attention of the assoeiation to the fact that this subject embraced a wide field. The hygienic treatment of mothers during gestation should not be overlooked.

Dr. Sandusky related the case of Mr. P——, aet. sixty-four, occupation farmer, who had for years been suffering with neuralgia of acute form in fifth pair of nerves; had extracted all the teeth but as yet had obtained no relief. The patient was exhibited before the association.

Subject passed.

The President's paper* was read and made the subject for discussion at 10 o'clock Friday morning.

Operative Dentistry was the next subject for discussion.

Dr. Freeman said we had almost ceased to pride ourselves on mechanical dentistry, Here the field of operative dentistry covers almost everything. We take pride in our profession. Many things in our every day practice is new to our brethren, and will be of much interest to some one. Here we must speak freely.

Dr. Morgan I am more impressed every day of my life with the worthlessness of what are called cheap fillings. I hold no feeling in common with the theory of electric disintegration. Am better satisfied every day of my life with the

*See Dental Register, November, 1878, p. 453.

excellence of cohesive gold. I think it is the acme of perfection.

Dr. Freeman said he was not an extremest. Is surprised at the assertion that the best operators use almost exclusively cohesive gold. The general demand of the public is the main cause of our catering to the demand for cheap fillings, hence the use of soft gold and plastic fillings. He read from his notes some of his recorded cases of treatment of dead and exposed nerves.

AFTERNOON.

Operative dentistry resumed.

Dr. Chisholm—I think I am an impartial man, and I occupy a middle ground, having almost gone out of the profession, I speak fully my convictions. I am clearly of the opinion, after a practice of thirty-five or forty years, that the best fillings that are, or ever have been made, were made of soft foil. The best men in the profession all over the world are abandoning the use of cohesive gold. He cited a case and challenged a demand for exhibition where cohesive gold had been used to the tune of one hundred and seventy dollars, which, after three months proved a complete failure. I claim that in this case the greatest amount of good would have been secured by filling these teeth with amalgam. We should be conservative in our treatment, and the teeth of our patients, and treat them as we would be treated. I never kill a pulp and if I can not preserve it I extract the tooth. Every dead tooth results in abscess. I use the term hard and soft foil.

Dr. Morgan—I do not use hard foil.

Dr. Chisholm—The soft foil that makes hard plugs and decays the teeth I object to, but the hard foil that makes soft fillings and saves the teeth I approve,

Dr. Morgan—I know that many men claim that gold is not the best material for filling teeth, but yet the best material in the hands of some men to fill teeth with. The case referred to by Dr. Chisholm is one of those isolated cases where any treatment in the world might have failed, but if gold is properly manipulated, it is the best filling for the teeth ever used for that purpose.

Dr. Freeman related a case in which, owing to financial disability of patient, the teeth were filled with amalgam in part. Afterwards had the same teeth among others filled with gold and gave much better satisfaction. It is true our forefathers used non-cohesive gold and saved teeth for scores of years, but they never filled badly decayed teeth, but small and favorable cavities. The failures of the present day are of a difficult kind.

Dr. Cobb—I would like to make an emphatic speech on this subject. I endorse nearly all Dr. Chisholm said, but in reference to devitalized teeth he is mistaken. A great many dead teeth have abscesses, but admitting that many of them do fail, much good results from saving them.

I do destroy nerves and fill the teeth, and have but few failures. I give Dr. King the credit of introducing the method of c ipping nerves.

I claim that the better class of fillings that are made to-day are made of non-cohesive gold. A retrospect of the work of the past proves this assertion.

Cohesive gold clogs, works stubborn and when packed has a tendency to roll or condense to the center of the cavity.

Non-cohesive spreads to the walls and stops out the fluids, and preserves the tooth.

Cohesive gold is good for contour work, but if we teach our young men how to fill teeth with non-cohesive gold, better results would be secured, and as the dentists of my age are passing away let me say to you, learn to fill teeth with non-cohesive gold.

I hope to see the day when for cheap fillings we will have a cement that will preserve teeth. I know some men will frown down anything that can be done cheaply. If we can get anything that will enable us to fill ten teeth for the same we can fill one for, it is better for us and better for our patients.

Dr. Sandusky learned the old method of filling with non-cohesive gold and still uses it. Am free to say that the most I have learned about working gold I learned in this body. Have gradually gotten to filling teeth with cohesive gold. I

believe it is perfection of the work that saves the teeth and not what kind of gold is used.

Nine-tenths of all the dentists who fill teeth through the country can save more teeth with amalgam than gold, because they are unable to make a good filling of anything else.

Dr. Morgan called attention to the subject of Oral Electricity, as published in the Missouri Dental Journal for June. Among fillings running under the gum or coming in contact with the gums there will be electric disintegration.

I dissent from Dr. Chisholm's treatment of devitalizing pulps. Have not as much confidence in capping pulps as formerly. I will endorse all he said about using Oil of Cloves in place of Creosote. Tin, gold or lead may be successfully used in filling roots of dead teeth—vegetable matter will not do. In straight rooted teeth I prefer to put in a gold wire, after the plan of Mack's screws.

Dr. Chisholm described his method of filling condemned teeth so as to prevent oxidization, viz: fill as full as you can with oxy-chloride, and then fill with gold or amalgam.

Believes a large amount of failures result from too much malleting or packing. Gold will pack upon itself until you loosen the adaptation to the walls of the tooth. Believes a better filling can be made by hand pressure alone—by hand packing—than by too much malleting.

Dr. Morgan said the objection to the use of cohesive gold is, that its opponents are unable to use cohesive gold with skill.

Dr. Cobb—Dr. Morgan hit the nail on the head and I deny that any man in the profession has the skill to make perfect fillings in all classes of cavities with cohesive gold. Four-fifths of the cavities can not be filled as well with cohesive as non-cohesive gold. Take all the teeth that have been filled in the ten years of cohesive gold, you find a greater number of failures than the ten years preceeding, when non-cohesive gold was used. He asked for the statistics of the average length of time gold fillings preserve teeth.

Dr. Freeman—I do not recognize proficiency in any slip-shod way of doing things, if it requires more skill to make a cohesive filling than a non-cohesive one, strive to attain to the highest skill.

Dr. Chisholm—It is the striving for skill that has brought me to where I stand, but when I see failure after failure from the use of cohesive gold, I think that all these men that claim the skill are the very men that are making the greatest failures.

Dr. Russell said Dr. Chisholm had struck the key note of success in the profession. It is the country dentist who does not try to build up large heavy contour fillings, mansard roofs, that, if we please, save teeth; therefore I do not, as Dr· Freeman does, undertake to instruct them. I believe the time is coming when we will do away with gold altogether, and I hope the time is not far off when the day will dawn.

I know that the best and most durable work now in existence is make of Abby's old-fashioned non-cohesive foil, and I do hope that the Lord will throw light on the minds of our skillful hard foil fillers, and bring them back to their old ways, so they can save teeth.

Honesty and competency is all that is necessary to make a good dentist. Be practical, be honest, be faithful, and save teeth the best you can, and do not promise too much.

Dr. Cobb—How long do a majority of fillings last?

Dr. Russell—I think cohesive fillings last on an average about three years; with soft foil the average is ten years.

Dr. Sandusky—We often go to extremes in our discussions. We should confine ourselves to reason, and be governed by rational views in filling teeth. In answer to a question of Dr. Cobb, Dr. Sandusky said his fillings had lasted thirteen years. How much longer they would last he did not know. Dr. Morgan said out of one hundred and seventy fillings not one had been lost in seventeen years. I believe that a majority of my fillings are buried with my patients. The average duration of amalgam fillings is less than five years, but it is because it is used in frail teeth. The average of gold about ten years.

Dr. Chisholm said the average duration of a successful filling was about ten years, and that of amalgam in the same teeth will average not two years less.

Dr. Cobb—Gold fillings will preserve teeth about ten years. And amalgam from five to seven, under unfavorable circumstances.

Dr. Crawford asked of Dr. Morgan, how long is the longest time a fifty grain gold filling has lasted?

Dr. Morgan—Twenty-one or twenty-two years.

Dr. Beach said, in the heat of discussion men seem to loose sight of the fact that good fillings are made of almost all materials. Am glad to hear Dr. Morgan say he sometimes uses semi or non-cohesive gold. The best interest of our patrons demands that the best means at our command be adopted to secure the ends we have in view. Some teeth can be best filled with cohesive and some with non-cohesive gold, and he who pins his practice down to the exclusive use of either, deprives himself of some of the very best means of saving teeth and his patients. I dare say of the very teeth they are seeking to save. One of the very very best materials for filling teeth has been left out of this discussion altogether, that is tin. All cavities not exposed directly to the friction of mastication are better preserved by tin than any other known metal. Let who will deny it, it is a fact that I stand ready to prove by actual demonstration, in the mouth of any patient who may be introduced by any one who doubts this assertion. Lead is the best and only metal fit for filling roots of dead teeth. Vegetable matter will not do. In answer to Dr. Cobb, Dr. Beach said the average gold fillings taking all operators into count was ten years, but the average of the best operators was much higher. Amalgam in the same hands and teeth will average less, how much he does not know.

Dr. Ross—The majority of gold fillings will last ten to fifteen years.

Subject passed.

(CONTINUED IN NEXT NUMBER.)

